# Cardiovascular outcomes in patients with locally advanced and metastatic prostate cancer treated with luteinising-hormone-releasing-hormone agonists or transdermal oestrogen: the randomised, phase 2 MRC PATCH trial (PR09)

**DOI:** 10.1016/S1470-2045(13)70025-1

**Published:** 2013-04

**Authors:** Ruth E Langley, Fay H Cafferty, Abdulla A Alhasso, Stuart D Rosen, Subramanian Kanaga Sundaram, Suzanne C Freeman, Philip Pollock, Rachel C Jinks, Ian F Godsland, Roger Kockelbergh, Noel W Clarke, Howard G Kynaston, Mahesh KB Parmar, Paul D Abel

**Affiliations:** a MRC Clinical Trials Unit, London, UK; b Brighton and Sussex University Hospitals NHS Trust, Royal Sussex County Hospital, Brighton, UK; c Beatson West of Scotland Cancer Centre, Glasgow, UK; d National Heart and Lung Institute, Imperial College London, Ealing and Royal Brompton Hospitals, London, UK; e Mid Yorkshire NHS Trust, Wakefield, UK; f Cancer Research UK and UCL Cancer Trials Centre, University College London, London, UK; g Diabetes Endocrinology and Metabolic Medicine, Imperial College London, London, UK; h Department of Urology, Leicester General Hospital, University Hospitals of Leicester, Leicester, UK; i The Christie NHS Foundation Trust and Salford Royal NHS Trust, Salford, UK; j Department of Surgery, Cardiff School of Medicine, Cardiff, UK; k Department of Surgery, Hammersmith Hospital, Imperial College London and Imperial College NHS Trust, London, UK

## Abstract

**Background:**

Luteinising-hormone-releasing-hormone agonists (LHRHa) to treat prostate cancer are associated with long-term toxic effects, including osteoporosis. Use of parenteral oestrogen could avoid the long-term complications associated with LHRHa and the thromboembolic complications associated with oral oestrogen.

**Methods:**

In this multicentre, open-label, randomised, phase 2 trial, we enrolled men with locally advanced or metastatic prostate cancer scheduled to start indefinite hormone therapy. Randomisation was by minimisation, in a 2:1 ratio, to four self-administered oestrogen patches (100 μg per 24 h) changed twice weekly or LHRHa given according to local practice. After castrate testosterone concentrations were reached (1·7 nmol/L or lower) men received three oestrogen patches changed twice weekly. The primary outcome, cardiovascular morbidity and mortality, was analysed by modified intention to treat and by therapy at the time of the event to account for treatment crossover in cases of disease progression. This study is registered with ClinicalTrials.gov, number NCT00303784.

**Findings:**

85 patients were randomly assigned to receive LHRHa and 169 to receive oestrogen patches. All 85 patients started LHRHa, and 168 started oestrogen patches. At 3 months, 70 (93%) of 75 receiving LHRHa and 111 (92%) of 121 receiving oestrogen had achieved castrate testosterone concentrations. After a median follow-up of 19 months (IQR 12–31), 24 cardiovascular events were reported, six events in six (7·1%) men in the LHRHa group (95% CI 2·7–14·9) and 18 events in 17 (10·1%) men in the oestrogen-patch group (6·0–15·6). Nine (50%) of 18 events in the oestrogen group occurred after crossover to LHRHa. Mean 12-month changes in fasting glucose concentrations were 0·33 mmol/L (5·5%) in the LHRHa group and −0·16 mmol/L (−2·4%) in the oestrogen-patch group (p=0·004), and for fasting cholesterol were 0·20 mmol/L (4·1%) and −0·23 mmol/L (−3·3%), respectively (p<0·0001). Other adverse events reported by 6 months included gynaecomastia (15 [19%] of 78 patients in the LHRHa group *vs* 104 [75%] of 138 in the oestrogen-patch group), hot flushes (44 [56%] *vs* 35 [25%]), and dermatological problems (10 [13%] *vs* 58 [42%]).

**Interpretation:**

Parenteral oestrogen could be a potential alternative to LHRHa in management of prostate cancer if efficacy is confirmed. On the basis of our findings, enrolment in the PATCH trial has been extended, with a primary outcome of progression-free survival.

**Funding:**

Cancer Research UK, MRC Clinical Trials Unit.

## Introduction

Around 900 000 men worldwide are diagnosed as having prostate cancer every year,^1^ and more than 40% receive androgen-deprivation therapy (ADT) within 6 months of diagnosis.^2^ ADT is the standard treatment for metastatic disease, but is also used at earlier stages as adjuvant and neoadjuvant therapy in men who require radical treatment for localised disease and for those with rising concentrations of prostate-specific antigen (PSA) who are at high risk of distant metastases.^3^

The immediate effects of castration are loss of male secondary sexual characteristics, impotence, muscle weakness, and changes in body composition.^4^

ADT is most frequently achieved with luteinising-hormone-releasing-hormone agonists (LHRHa), but these drugs are associated with long-term toxic effects, including decreased bone-mineral density,^5^, ^6^ osteoporotic fractures,^7^, ^8^, ^9^, ^10^ adverse metabolic changes,^11^ and diabetes.^10^, ^12^, ^13^ Whether this treatment leads to cardiovascular complications is unclear because evidence is inconsistent. A joint advisory statement from the American Heart Association, American Cancer Society, and American Urological Association, while noting the inconsistencies in the data, advocates careful management of cardiac disease in patients with prostate cancer treated with ADT.^14^ Additionally, the US Food and Drug Administration requires LHRHa drug labels to warn of an increased risk of diabetes and cardiovascular disease.

Continuous exposure to LHRHa leads to downregulation of pituitary receptors and hormones, and, subsequently, castrate concentrations of testosterone. Oestrogens in men are derived from the aromatisation of androgens and, therefore, LHRHa also reduce oestrogen concentrations. Thus, men can develop toxic effects related to low concentrations of these two sex hormones.^4^ The use of oestrogen is an alternative approach to ADT in men. It decreases testosterone concentrations in serum by inhibition of the hypothalamic-pituitary axis but, unlike LHRHa, is not associated with toxic effects related to oestrogen depletion. Oral oestrogen (eg, diethylstilbestrol) was used for ADT before the development of LHRHa, but is no longer used routinely because it is associated with an increased risk of thrombotic complications^15^ attributed to the effects of first-pass hepatic metabolism on coagulation proteins and lipids.^16^ Administration of oestrogen parenterally (intravenously, intramuscularly, or transcutaneously) avoids first-pass hepatic metabolism and, therefore, is not expected to be associated with the same thrombotic complications as oral oestrogen.^16^, ^17^ Parenteral oestrogen could, therefore, be a potential therapeutic alternative to LHRHa. We did the Prostate Adenocarcinoma: TransCutaneous Hormones versus luteinising hormone-releasing hormone agonists (PATCH) randomised, phase 2 trial to assess the safety and activity of transdermal oestrogen patches in the treatment of prostate cancer.

## Methods

### Patients

Eligible men had locally advanced or metastatic prostate cancer (including those previously treated with radical intent with rising concentrations of PSA) and scheduled to start continuous indefinite hormone therapy at multiple hospitals in the UK. They were also required to have testosterone concentrations of 6·0 nmol/L or higher and a WHO performance status score of 0–2. Administration of radical radiotherapy to the prostate was not initially permitted, but was later allowed to reflect changes in practice. All patients underwent electrocardiography and chest radiography. Echocardiography was added to baseline investigations if a patient had a history of ischaemic heart disease. Other baseline investigations were physical examinations (including blood pressure), bone scans, CT or MRI at the discretion of the physician, and blood tests, including measurement of PSA concentration. Cardiovascular exclusion criteria were stroke or transient ischaemic attack within the previous 2 years; radiologically confirmed deep-vein thrombosis or pulmonary embolism at any time; myocardial infarction in the 6 months before the study or more than 6 months previously with evidence of q-wave infarction on electrocardiography at screening; angina (New York Heart Association grade III or higher) in the previous year; symptoms of heart failure (New York Heart Association grade III or higher); pulmonary oedema evident on chest radiography at screening; left-ventricular ejection fraction of 40% or lower in patients with a history of ischaemic heart disease or heart failure; and systolic blood pressure of 160 mmHg or higher and, diastolic blood pressure of 100 mmHg or higher, or both, in men. Other exclusion criteria were previous systemic therapy or previous or current malignant disease or cardiovascular disease thought likely to compromise the patient's ability to tolerate therapy or affect assessment.

The protocol was approved by national regulatory and ethics committees and participating hospitals obtained the appropriate local approvals. Participants provided written informed consent.

### Randomisation

Men were allocated in a 2:1 ratio to receive oestrogen patches or LHRHa. Randomisation was done centrally at the trials unit, according to a computer-based minimisation algorithm with a random element (80%) balanced for the following factors: disease stage, age, smoking status, personal or family history of heart disease, and which LHRHa agent was to be used. Staff at the study centres contacted the trials unit by telephone to obtain allocation details. The trial was open-label, but primary outcome events were reviewed by an independent endpoint review committee that was unaware of treatment allocation.

### Procedures

Initially, patients in the oestrogen-patch group received three patches (100 μg per 24 h) to be self-administered and changed twice weekly for 4 weeks. The number of patches was reduced to two twice weekly if castrate testosterone concentrations in serum of 1·7 nmol/L or lower were achieved (regimen one). This biweekly regimen was based on previous data^18^ and was intended to be practical and to achieve castrate testosterone concentrations quickly and maintain them. The first review by the independent data monitoring committee showed that oestradiol concentrations were lower and testosterone responses were less frequent than anticipated. The regimen was changed, therefore, to four patches to be changed twice weekly for 4 weeks, followed by use of three patches twice weekly when the target castrate testosterone concentration in serum was reached (regimen two).^19^ Patients with testosterone concentrations higher than castrate levels at 4 weeks remained on the induction regimen and had testosterone checked every 2 weeks. LHRHa was prescribed according to local practice and could be accompanied by a short course of antiandrogens to treat tumour flare. Testosterone and PSA concentrations were checked regularly in all participants. In cases of disease progression, patients could be given second-line therapy at the discretion of the treating clinician, including changing to the non-assigned study treatment.

The primary outcome measure was cardiovascular morbidity and mortality, according to the following prespecified definitions: new symptoms or clinical signs of decompensated cardiac failure, supported by chest radiography, echocardiography, or a rise in concentration of brain natriuretic peptide; acute coronary syndrome (including unstable angina, non-ST-segment-elevation myocardial infarction, and myocardial infarction) presenting as new-onset cardiac chest pain, collapse, or shortness of breath, and confirmed as ischaemic in origin by electrocardiography, troponin rise, coronary angiography, or a combination of these tests; new neurological symptoms and signs of a cerebrovascular accident, confirmed by brain CT or MRI or by clinical diagnosis and carotid duplex scanning for transient ischaemic attacks, with evidence of pre-existing or new, persistent or paroxysmal atrial fibrillation; new clinical symptoms supported by radiological evidence of other arterial embolic events; venous thromboembolism confirmed by ultrasonography, venography, or both, or pulmonary embolism confirmed by CT pulmonary angiogram, ventilation-perfusion scans, or angiography; and other relevant events (deaths attributed to one of these cardiovascular causes without supporting documentation, or events that did not meet the exact definitions but were judged appropriate for inclusion by the independent reviewers).

Cardiac events were reported by investigators at 3 and 6 months and 6-monthly thereafter on a specifically designed form or identified from reports of serious adverse events and routinely collected data on toxic effects. Two independent reviewers unaware of original treatment allocation or current treatment being received reviewed original documentation and decided which events met the primary outcome definitions and whether or not they were related to hormone therapy. Discrepancies in classification were resolved by discussion, assessment of further clinical information, or both.

Secondary outcomes were hormone responses and other adverse events, including changes in cardiovascular risk factors (concentrations of fasting glucose, fasting total cholesterol, and HDL cholesterol, weight, and blood pressure) and other toxic effects. Severity of events was assigned according to the Common Terminology Criteria for Adverse Events (version 3.0). Laboratory assessments of hormones and metabolic factors were done in the participating hospitals without central validation.

### Statistical analysis

We estimated that around 8% of men receiving LHRHa would have a cardiovascular event, on the basis of previous data.^20^ As a phase 2 trial, the analysis was not powered to compare arms. A sample size of 200 patients, of whom 133 would be randomised to receive oestrogen patches, was calculated to be large enough to estimate the cardiovascular event rate in the oestrogen-patch group with reasonable precision. The LHRHa group acted as a benchmark in view of the uncertainty about the underlying cardiovascular risk in the cohort. The 2:1 randomisation ratio was designed to maximise experience with oestrogen patches. After regimen two was introduced in the oestrogen-patch group, the required sample size was increased to 250 patients overall (133 to regimen two). If strong evidence of a 15% or higher cardiovascular-event rate in the oestrogen-patch arm was seen (based on the lower limit of a 95% CI), an early review by the independent data monitoring committee would be triggered and trial closure could be considered. The numbers of events required to trigger a review were ten or more in the first 33 patients assigned oestrogen patches, 17 in 67 patients, 23 in 100 patients, and 29 in 133 patients.

The primary cardiovascular analysis was based on modified intention-to-treat principles: patients were assessed in the oestrogen-patch group if they had been treated with patches at any point, and assessed in the LHRHa group if they had received LHRHa but not patches at any point; patients who received no treatment at all were excluded. This approach was expected to be conservative because it maximised the number of events attributed to oestrogen patches. The proportion of patients who experienced an event is reported with exact binomial 95% CIs. As crossover was allowed in the case of disease progression, we did a planned sensitivity analysis in which we included numbers of events according to treatment being taken at the time of (or within 30 days before) the events. Analysis of the primary outcome was planned for 3 months after the last randomisation to enable a prompt decision regarding continuation of the study, whereas assessment of the secondary outcomes were done 15 months after the last randomisation to enable assessment of changes over time. Secondary outcomes were assessed in men who were still receiving their assigned study treatment with no additional prostate-cancer therapy at the time of interest. Men with oestradiol concentrations of 250 pmol/L or lower, or who were reported to have stopped using patches (where oestradiol data were not available) were not assessed. Castration rates (proportion of men with testosterone concentrations of 1·7 nmol/L or lower) at 3 months and 6 months are reported. Post-hoc analyses included the proportions of patients who achieved lower testosterone thresholds (1·1 mmol/L and 0·7 nmol/L) and differences between LHRHas. Cardiovascular risk factors are reported as mean (95% CI) at 6 and 12 months, as well as mean (%) changes from baseline. Treatment effects were investigated with ANCOVA models adjusted for baseline values. Checks of model assumptions and fit included assessment of residual plots and tests. We did no formal treatment comparisons of toxic effects. All analyses were done with Stata statistical software (version 12).

In this study, we report data from the preplanned analysis of cardiovascular outcomes in the first 254 patients. On the basis of recommendations from the independent data monitoring committee, the study was extended to assess progression-free survival in 660 men (including this initial cohort). To maintain the integrity of the ongoing study, data on disease status during follow-up are not presented.

The trial is registered with ISRCTN Register and ClinicalTrials.gov, numbers ISRCTN70406718 and NCT00303784.

### Role of the funding source

The sponsor of the study had no role in the study design, data collection, data analysis, data interpretation, or writing of the report. REL, FHC, SCF, and RCJ had access to the raw data, and processed data released by the independent data monitoring committee were available to all authors. REL, FHC, and PDA were jointly responsible for the decision to submit for publication.

## Results

**Figure 1:**
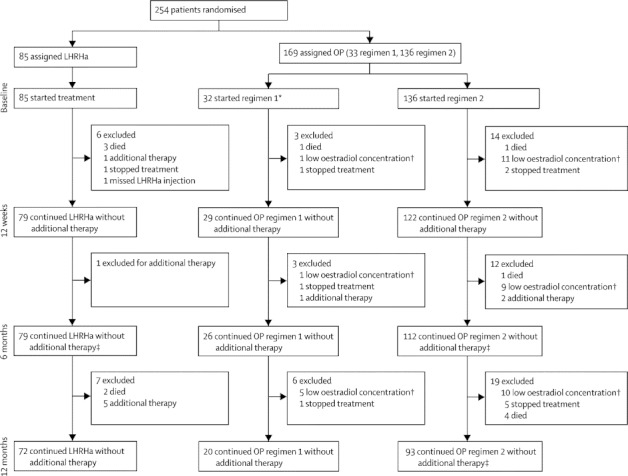
Trial profile Randomisation was 2:1 (oestrogen patches:LHRHa). LHRHa=luteinising-hormone-releasing-hormone agonists. OP=oestrogen patches. *One patient did not start treatment, withdrew soon after randomisation, and did not attend any trial visits. †Men with oestradiol concentraions of 250 pmol/L or lower were assumed not to be using OP and were excluded. ‡Includes a small number of individuals (maximum 3) who had been assumed not to be receiving treatment at an earlier time (eg, owing to low oestradiol concentrations, reported difficulty in using OP, or missed appointments).

Between April 7, 2006, and April 28, 2010, 254 patients from 27 UK centres were enrolled, 169 into the oestrogen-patch group (33 regimen one, 136 regimen two) and 85 into the LHRHa group. With the exception of one patient assigned to oestrogen patches, all men started their assigned treatment (figure 1). Characteristics at randomisation were much the same between groups (table 1). Median age was 74 years (IQR 69–79), 91 (36%) patients had metastatic disease, 236 (93%) had WHO performance status scores of 0 or 1, 142 (56%) were current or previous smokers, and 70 (28%) were long-term regular aspirin users. Five (2%) men had undergone radical radiotherapy (three in the oestrogen-patches group and two in the LHRHa group).

**Table 1:** Baseline characteristics of the study population by allocated treatment

		**LHRHa (n=85)**	**OP (n=169)**
Age at randomisation (years)			
	Median (IQR)	75 (69–80)	73 (69–78)
	Range	56–92	49–90
PSA concentration (ng/mL)			
	Median (IQR)	36 (19–106)	55 (21–153)
	<50	53 (62%)	78 (46%)
	50–500	28 (33%)	75 (45%)
	>500	4 (5%)	15 (9%)
	Unknown	0	1
Gleason sum score			
	4–6	10 (13%)	16 (10%)
	7	33 (42%)	60 (38%)
	8–10	35 (45%)	83 (52%)
	Unknown	7	10
T category			
	T0/X	7 (8%)	11 (7%)
	T1/2	5 (6%)	10 (6%)
	T3	59 (69%)	129 (76%)
	T4	14 (16%)	19 (11%)
N category			
	N0	28 (33%)	43 (25%)
	N+	17 (20%)	36 (21%)
	NX	40 (47%)	90 (53%)
M category			
	M0	53 (62%)	110 (65%)
	M1	32 (38%)	59 (35%)
Regular long-term aspirin use		24 (28%)	46 (27%)
Smoking history			
	Never	38 (45%)	74 (44%)
	Previous	37 (44%)	72 (43%)
	Current	10 (12%)	23 (14%)
WHO performance status			
	Normal activity	56 (66%)	118 (70%)
	Avoid strenuous activity	22 (26%)	40 (24%)
	Up and about >50%	7 (8%)	11 (7%)
Mean (SD, n) fasting cholesterol concentration (mmol/L)		5·0 (1·2, n=80)	4·7 (1·1, n=162)
Mean (SD, n) fasting glucose (mmol/L)		5·7 (1·0, n=81)	5·7 (1·2, n=161)
Mean (SD, n) weight (kg)		80·1 (12·9, n=77)	82·0 (13·1, n=153)
Mean (SD, n) blood pressure (mm Hg)			
	Systolic	144 (14·9, n=80)	140 (15·3, n=165)
	Diastolic	79 (10·2, n=80)	79 (9·2, n=165)

Data are n (%) unless otherwise indicated. LHRHa=luteinising-hormone-releasing hormone agonists. OP=oestrogen patches.

At 3 months, among men who were still receiving their allocated treatment without additional therapy, 70 (93%) of 75 with data available in the LHRHa group and 111 (92%) of 121 receiving oestrogen patches under regimen two had testosterone concentrations of 1·7 nmol/L or less. By contrast, 20 (69%) of 29 receiving oestrogen patches under regimen one had castrate concentrations of testosterone. The castration rate in the LHRHa group did not differ by agonist used (28 [93%] of 30 for leuprorelin, 41 [93%] of 44 for goserelin, and one [100%] of one for triptorelin). Testosterone concentrations of 1·1 mmol/L or lower were seen in 53 (71%) of 75, 94 (78%) of 121, and 14 (48%) of 29 men who received LHRHa, oestrogen-patch regimen two, and oestrogen-patch regimen one, respectively, and concentrations of 0·7 nmol/L or lower were seen in 44 (59%) of 75, 75 (62%) of 121, and nine (31%) of 29.

At 6 months, 68 (88%) of 77 patients with available data had testosterone concentrations of 1·7 nmol/L or lower in the LHRHa group, compared with 106 (95%) of 112 in the oestrogen-patch group receiving regimen two and 20 (77%) of 26 of those receiving regimen one. Testosterone breakthrough was seen at 6 months in six (9%) of 68 in the LHRHa group and four (4%) of 104 in the oestrogen-patch group (regimen two) who had had castrate concentrations at 3 months. Oestradiol concentrations higher than 250 pmol/L indicated that adherence to treatment with oestrogen patches was generally good, and at 3 months most (117 [96%] of 122) men randomised to receive oestrogen-patch regimen two had changed to the maintenance regimen of three patches; two (2%) were using two patches and three (2%) were using four patches.

Median follow-up for cardiovascular events was 19 months (IQR 12–31, minimum 3 months). 55 potential events were identified among patients in the modified intention-to-treat cohort, of which 24 met the outcome definitions (table 2). The number of patients with an event in the LHRHa group was six (7·1%) of 84 (95% CI 2·7–14·9, six events) and in the oestrogen-patches group was 17 (10·1%) of 169 (6·0–15·6, 18 events). The number of events in the oestrogen-patch group remained lower than the predefined criteria for early review by the independent data monitoring committee throughout the trial. The rate of cardiovascular events was 2·9% higher in the oestrogen-patch group than in the LHRHa group (95% CI −4·2 to 10·1). The wide 95% CI suggests no significant difference between groups, although the trial was not designed to test this comparison.

**Table 2:** Cardiovascular events

			Intention-to-treat analysis*		**Treatment at time of event**†	
			LHRHa (n=84)	OP (n=169)	LHRHa	OP
Cardiovascular events						
	Number of events (fatal events)		6 (1)	18 (5)	15 (4)	9 (2)
	Number of patients		6 (7%)	17 (10%)	15‡	9‡
	Type of event (fatal events)					
		Heart failure	0	3 (1)	3 (1)	0
		Acute coronary syndrome	2 (1)	6 (1)	5 (2)	3 (0)
		Thromboembolic stroke	1 (0)	4 (1)	2 (0)	3 (1)
		Other arterial embolic events	0	1 (1)	0	1 (1)
		Venous thromboembolism	3 (0)	4 (1)	5 (1)	2 (0)
	Rate of events per 100 patients		7·1	10·7	NA	NA
	Proportion (exact binomial 95% CI) of patients with events (%)		7·1% (2·7–14·9)	10·1% (6·0–15·6)	NA	NA
Total reviewed events that did not satisfy outcome definitions			10	21	14	17

LHRHa=luteinising-hormone-releasing-hormone agonists. OP=oestrogen patches.

* In the modified intention-to-treat population, patients were included in the OP group if they had been treated with OP at any point; patients were included in the LHRHa group if they had been treated with an LHRHa and had not received OP at any point. One patient assigned to LHRHa who received some OP treatment was included in the OP group, and one assigned to OP who received no treatment was excluded.

† Events within 30 days of changing treatments were assigned to the original treatment.

‡ One patient had two events, one while using OP and one while taking LHRHa, and is included in both columns.

Of the 18 events among men assigned oestrogen patches, nine (50%) occurred more than 30 days (and in four cases more than 12 months) after oestrogen was stopped and LHRHa was started. 13 (72%) of the 18 events in the oestrogen-patch group and four (67%) of the six in the LHRHa group were deemed possibly related to the allocated trial treatment by one or both independent reviewers. These outcome events included six in the oestrogen-patch group that occurred more than 30 days after the patients had switched to LHRHa. In the oestrogen-patch group, 13 of the 18 cardiovascular events occurred in 12 men who were receiving regimen two.

Six (25%) of the 24 cardiovascular events were fatal, of which three were thought to be possibly related to the study treatment by the independent reviewers: a thromboembolic stroke in a man assigned to oestrogen patches and using them at the time of the event (36 months); a myocardial infarction in a man assigned to LHRHa and receiving it at the time of the event (2 months); and a pulmonary embolism in a man assigned to oestrogen patches who had switched to LHRHa 16 months before death. One further death potentially related to treatment was reported up to July, 2011—an acute myocardial infarction in a patient who was assigned to LHRHa and had continued to take that treatment until death (33 months).

31 events were deemed not to meet the primary-outcome definitions: non-cardiac chest pain or investigation for a silent myocardial infarction that was not confirmed (n=6); symptoms that might indicate congestive cardiac failure or venous thromboembolism, such as dyspnoea or leg swelling, but for which the causes were not confirmed (n=5); other cardiac events, including atrial fibrillation, hypotension, hypertension, and non-embolic peripheral vascular disease (n=11); other medical events (n=4); and symptoms associated with an outcome event that did not constitute a separate event (n=5).

**Figure 2:**
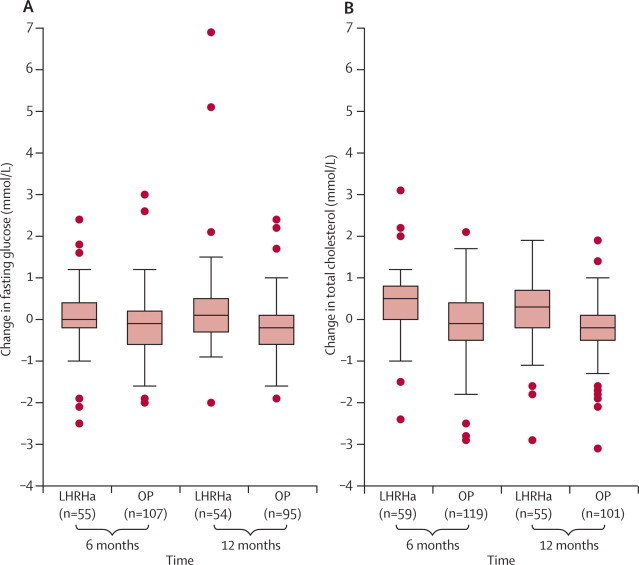
Changes in fasting glucose (A) and total cholesterol (B) concentrations in patients still receiving treatment at 6 and 12 months Patients were not receiving additional therapy. Boxes indicate median and IQR, whiskers indicate 1·5×IQR, and dots indicate outlying values. LHRHa=luteinising-hormone-releasing-hormone agonists. OP=oestrogen patches.

In men who were still receiving the allocated treatment without additional therapy at 6 months, mean fasting glucose had increased in the LHRHa group and decreased in the oestrogen-patches group, which led to a significant difference between groups (table 3, figure 2). In men who remained on assigned treatments at 12 months, fasting glucose concentrations increased further in the LHRHa arm but remained similar in the oestrogen-patches group, with the mean changes from baseline being 0·33 mmol/L (5·5%) and −0·16 mmol/L (−2·4%), respectively (p=0·004).

**Table 3:** Cardiovascular risk factors in men who remained on randomised treatment without additional therapy

		**Number of patients**	**Mean (95% CI) at baseline**	**Mean (95% CI) at time of assessment**	**Mean change (range)**	**Mean change (%)**	Treatment effect p value*
**6 months**							
Fasting glucose (mmol/L)							
	LHRHa	55	5·76 (5·48–6·04)	5·82 (5·53–6·12)	0·06 (−2·5 to 2·4)	2·0%	0·035
	OP	107	5·59 (5·43–5·74)	5·44 (5·27–5·61)	−0·15 (−2·0 to 3·0)	−2·1%	
Fasting cholesterol (mmol/L)							
	LHRHa	59	4·99 (4·73–5·26)	5·35 (5·00–5·69)	0·35 (−2·4 to 3·1)	7·6%	<0·0001
	OP	119	4·81 (4·62–4·99)	4·68 (4·51–4·85)	−0·13 (−2·9 to 2·1)	−1·2%	
Fasting HDL (mmol/L)							
	LHRHa	58	1·30 (1·21–1·38)	1·41 (1·30–1·53)	0·12 (−0·5 to 1·4)	10·1%	0·35
	OP	108	1·29 (1·23–1·36)	1·45 (1·37–1·53)	0·16 (−0·6 to 1·1)	13·6%	
Weight (kg)							
	LHRHa	56	80·95 (77·35–84·55)	83·03 (79·30–86·76)	2·09 (−5·2 to 20·8)	2·7%	0·71
	OP	107	82·04 (79·56–84·53)	83·91 (81·29–86·53)	1·87 (−9·0 to 11·0)	2·3%	
Systolic blood pressure† (mm Hg)							
	LHRHa	60	144·0 (115·2–165·7)	142·0 (110·1–179·8)	0 (−39·0 to 50·0)	0	0·21
	OP	116	140·0 (113·0–160·6)	137·0 (110·0–172·2)	1·0 (−42·0 to 55·0)	−0·8%	
Diastolic blood pressure (mm Hg)							
	LHRHa	60	79·2 (76·7–81·7)	81·0 (78·0–84·0)	1·80 (−27·0 to 30·0)	2·9%	0·011
	OP	116	78·8 (77·2–80·3)	76·6 (74·5–78·7)	−2·18 (−31·0 to 27·0)	−2·4%	
**12 months**							
Fasting glucose (mmol/L)							
	LHRHa	54	5·70 (5·43–5·98)	6·03 (5·54–6·52)	0·33 (−2·0 to 6·9)	5·5%	0·004
	OP	95	5·50 (5·35–5·65)	5·34 (5·16–5·51)	−0·16 (−1·9 to 2·4)	−2·4%	
Fasting cholesterol (mmol/L)							
	LHRHa	55	5·11 (4·83–5·39)	5·31 (4·94–5·68)	0·20 (−2·9 to 1·9)	4·1%	<0·0001
	OP	101	4·79 (4·59–5·00)	4·56 (4·39–4·73)	−0·23 (−3·1 to 1·9)	−3·3%	
Fasting HDL (mmol/L)							
	LHRHa	52	1·29 (1·21–1·37)	1·36 (1·24–1·47)	0·07 (−0·9 to 0·8)	5·3%	0·33
	OP	91	1·27 (1·20–1·34)	1·38 (1·30–1·46)	0·11 (−0·3 to 1·0)	9·5%	
Weight (kg)							
	LHRHa	47	81·77 (77·92–85·61)	83·86 (79·61–88·11)	2·09 (−14·0 to 14·7)	2·5%	0·70
	OP	83	80·93 (78·06–83·81)	83·24 (80·10–86·38)	2·31 (−8·0 to 10·0)	2·7%	

LHRHa=luteinising-hormone-releasing-hormone agonists. OP=oestrogen patches.

* Tests for treatment effect are based on ANCOVA models with adjustment for baseline values.

† Owing to distribution, data are presented as median (5th and 95th percentiles) at baseline and 6 months, median change (range), and median percentage change. Log-transformed values were used for the ANCOVA model.

Mean fasting cholesterol concentration increased in the LHRHa group at 6 months, compared with a small decrease in recipients of oestrogen patches (table 3, figure 2). At 12 months, the mean value was similar to that at 6 months in the LHRHa group (5·31 mmol/L), but had decreased further in the oestrogen-patches group (4·56 mmol/L). Mean changes from baseline were 0·20 mmol/L (4·1%) in the LHRHa group and −0·23 mmol/L (−3·3%) in the oestrogen-patches group (p<0·0001). By contrast, HDL cholesterol concentrations had increased to a similar degree in the two groups at 6 months and 12 months (table 3).

Mean weight had increased by similar amounts in the two treatment groups at 6 months (table 3) and 12 months. Diastolic blood pressure had increased in the LHRHa group at 6 months and decreased in the oestrogen-patches group, but changes were small, as were changes in systolic blood pressure (table 3). These data were supported by those for adverse effects, with only a few reports being made of substantial changes in weight or blood pressure (table 4). Data on blood pressure were not collected at 12 months. Values for cardiovascular risk factors were similar after exclusion of patients randomised to regimen one of the oestrogen patches.

**Table 4:** Toxic effects reported up to 6 months in men who remained on assigned treatment without additional therapy

		LHRHa (n=78*)	**OP regimen one (n=26)**	**OP regimen two (n=112)**
**Endocrine/sexual**				
Gynaecomastia				
	0	63 (81%)	8 (31%)	26 (23%)
	1–2	15 (19%)	15 (57%)	76 (68%)
	3	0	3 (12%)	10 (9%)
Erectile dysfunction†				
	0	36 (47%)	9 (35%)	49 (45%)
	1–2	33 (43%)	15 (58%)	54 (49%)
	3	8 (10%)	2 (8%)	7 (6%)
Decreased libido‡				
	0	39 (51%)	8 (31%)	52 (47%)
	1–2	37 (49%)	18 (69%)	55 (50%)
	3	0	0	3 (3%)
Hot flushes				
	0	34 (44%)	18 (69%)	85 (76%)
	1–2	44 (56%)	8 (31%)	27 (24%)
**Dermatological**§				
Pruritis				
	0	74 (95%)	21 (81%)	82 (73%)
	1–2	4 (5%)	5 (19%)	30 (27%)
Erythema				
	0	77 (99%)	21 (81%)	86 (77%)
	1–2	1 (1%)	5 (19%)	26 (23%)
Eczema				
	0	76 (97%)	24 (92%)	105 (94%)
	1–2	2 (3%)	2 (8%)	7 (6%)
Urticaria				
	0	77 (99%)	24 (92%)	102 (91%)
	1–2	1 (1%)	2 (8%)	10 (9%)
Change in skin pigmentation				
	0	78 (100%)	24 (92%)	111 (99%)
	1–2	0	2 (8%)	1 (1%)
Hair changes				
	0	75 (96%)	26 (100%)	110 (98%)
	1–2	3 (4%)	0	2 (2%)
**Neurological**				
Anxiety				
	0	64 (82%)	24 (92%)	100 (89%)
	1–2	14 (18%)	2 (8%)	12 (11%)
Depression				
	0	64 (82%)	20 (77%)	101 (90%)
	1–2	13 (17%)	6 (23%)	11 (10%)
	3	1 (1%)	0	0
Inability to concentrate				
	0	62 (79%)	15 (58%)	104 (93%)
	1–2	16 (21%)	11 (42%)	8 (7%)
Increased irritability				
	0	62 (79%)	23 (88%)	101 (90%)
	1–2	16 (21%)	3 (12%)	11 (10%)
Headache				
	0	72 (92%)	25 (96%)	108 (96%)
	1–2	6 (8%)	1 (4%)	4 (4%)
Paraesthesia				
	0	77 (99%)	25 (96%)	111 (99%)
	1–2	1 (1%)	1 (4%)	1 (1%)
Dizziness				
	0	68 (87%)	23 (88%)	107 (96%)
	1–2	9 (12%)	3 (12%)	5 (4%)
	3	1 (1%)	0	0
**Gastrointestinal**				
Nausea				
	0	74 (95%)	24 (92%)	109 (97%)
	1–2	4 (5%)	2 (8%)	3 (3%)
Vomiting				
	0	76 (97%)	26 (100%)	111 (99%)
	1–2	2 (3%)	0	1 (1%)
Abdominal pain				
	0	71 (91%)	24 (92%)	112 (100%)
	1–2	7 (9%)	2 (8%)	0
Dyspepia				
	0	73 (94%)	25 (96%)	109 (97%)
	1–2	5 (6%)	1 (4%)	3 (3%)
**Constitutional and other symptoms**				
Fatigue				
	0	46 (59%)	16 (62%)	85 (76%)
	1–2	32 (41%)	10 (38%)	26 (23%)
	3	0	0	1 (1%)
Insomnia				
	0	66 (85%)	20 (77%)	100 (89%)
	1–2	12 (15%)	6 (23%)	12 (11%)
	3	0	0	0
Appetite increase				
	0	69 (88%)	21 (81%)	106 (95%)
	1–2	9 (12%)	5 (19%)	6 (5%)
Weight changes				
	0	64 (82%)	23 (88%)	99 (88%)
	1–2	12 (15%)	3 (12%)	13 (12%)
	3	2 (3%)	0	0
Sweating				
	0	51 (65%)	21 (81%)	104 (93%)
	1–2	26 (33%)	5 (19%)	8 (7%)
	3	1 (1%)	0	0
Oedema				
	0	71 (91%)	22 (85%)	102 (91%)
	1–2	7 (9%)	4 (15%)	10 (9%)
Blood-pressure changes				
	0	72 (92%)	26 (100%)	109 (97%)
	1–2	5 (6%)	0	3 (3%)
	3	1 (1%)	0	0
Chest pain				
	0	73 (94%)	26 (100%)	110 (98%)
	1–2	4 (5%)	0	1 (1%)
	3	1 (1%)	0	1 (1%)
Other				
	0	55 (71%)	18 (69%)	89 (79%)
	1–2	21 (27%)	7 (27%)	22 (20%)
	3	2 (3%) (palpitations, hyperglycaemia)	1 (4%) (urinary retention)	1 (1%) (angioplasty)

LHRHa=luteinising-hormone-releasing-hormone agonists. OP=oestrogen patches.

* One LHRHa patient with no 6-month toxic-effect data available was excluded.

† Data were missing for three patients (one LHRHa, two OP regimen two).

‡ Data were missing for four patients (two LHRHa, two OP regimen two).

§ Rates of skin toxic effects given in the text indicate the numbers of men who reported one or more of these symptoms.

Other adverse events were largely as expected, and were generally mild in the two treatment groups (table 4). As anticipated, the most frequently reported symptoms at 6 months in men still receiving their allocated treatment were related to sexual function, and, in some cases were severe (table 4). Gynaecomastia was reported on both treatments, but was more frequently reported in the oestrogen-patch group than in the LHRHa group, and in a small number of cases was symptomatic. By contrast, hot flushes were more frequently reported for patients in the LHRHa group than in the oestrogen-patches group (table 4). Minor dermatological problems associated with use of oestrogen patches were reported for 58 (42%) of 138 men, compared with ten (13%) of 78 in the LHRHa group. In the two groups all reported symptoms were grade 1–2 and included pruritis, erythema, eczema, urticaria, change in skin pigmentation (oestrogen-patches group only), and hair changes (LHRHa group only). Some men reported more than one symptom. No grade 4 or 5 adverse events had been reported by 6 months and, with the exception of the cardiovascular events noted above, no other potentially treatment-related deaths had been reported in this cohort to July, 2011.

## Discussion

Our results show that parenteral oestrogen administered via patches can lead to castrate testosterone concentrations similar to those achieved with LHRHa in men with locally advanced and metastatic prostate cancer. These findings confirm the results of a small pilot study.^21^ In our study, which excluded patients with high baseline risks of cardiovascular events, the rate of cardiovascular complications in men receiving oestrogen patches was similar to that in men receiving LHRHa. Additionally, it was lower than rates observed with oral oestrogen by the Veterans Administration Cooperative Urological Research Group.^15^, ^22^ Our modified intention-to-treat analysis of cardiovascular events seems to have been conservative, because several events attributed to the oestrogen-patches group occurred in men who had received oestrogen therapy for only a short period, or who had stopped treatment for a long time before the event occurred, or both. The analysis according to treatment at the time of the event provides a more standard assessment of toxic effects. Data on disease progression and survival are not yet available because efficacy will be assessed in the extended trial.

An important strength of this study is the independent, masked review of cardiovascular events. Several large population-based studies have reported associations between treatment with LHRHa and increased incidence of fatal and non-fatal cardiovascular events,^12^, ^13^, ^23^, ^24^ but other studies have shown no such relation.^10^, ^25^ A meta-analysis of eight randomised trials involving 4141 patients with median follow-up durations of 7·6–13·2 years compared immediate versus no or delayed LHRHa treatment in men with non-metastatic prostate cancer. Cardiovascular mortality of 11% was reported and did not differ between groups (relative risk 0·93, 95% CI 0·79–1·10).^26^ Data were extracted from trial reports and were subject to inconsistencies in definitions of events and assessment procedures. Effects on non-fatal events, time to cardiovascular death, and potential differences relating to pre-existing cardiovascular disease were not assessed.

Use of parenteral oestrogens to treat prostate cancer has been reviewed previously (panel).^27^ Parenteral oestrogen administration should avoid the venous thrombotic risk associated with oral administration while providing the arterial benefits attributed to oestrogen.^28^ A series of trials in Scandinavia assessed use of intramuscular polyoestradiol. The largest randomised 910 patients with metastatic prostate cancer to receive intramuscular polyoestradiol or combined androgen deprivation with triptorelin plus flutamide or orchidectomy. Biochemical progression, cancer-specific survival, cardiac mortality, and overall mortality did not differ between treatment groups.^29^ Men were excluded on the basis of cardiovascular risk only if they had had myocardial or cerebral infarction within the preceding month. Cardiovascular morbidity was higher in both groups among those who had a history of major cardiovascular disease than in those who did not, but the difference was greater in the polyoestradiol group.^30^ We did not assess differences by pre-existing cardiovascular risk, but such analysis will be possible in the larger cohort of the extended trial.

Panel **Research in context**

**Systematic review**

In a systematic review, 20 randomised controlled trials of parenteral oestrogen in patients with prostate cancer were identified from electronic databases, including Medline, Embase, and the Cochrane Central Register of Controlled Trials, with no restrictions on language or publication date.^27^ Relevant published papers and internet resources, such as trial and national drug registries, were also searched. The review found no consistent evidence that parenteral oestrogen given at doses sufficient to produce castrate testosterone concentrations differed from luteinising-hormone-releasing-hormone agonists in terms of prostate-cancer, cardiovascular, or overall mortality. Transdermal oestrogen patches had shown promise in terms of activity and toxic-effect profiles in the treatment of locally advanced or metastatic prostate cancer in a single-arm pilot study.^21^

**Interpretation**

Our study provides evidence that castrate testosterone concentrations similar to those seen in patients taking luteinising-hormone-releasing-hormone agonists can be achieved with transdermal oestrogen. Rates of cardiovascular toxic effects were similar with the two treatments and were lower than those seen with oral oestrogen.^15^ The study also provides data on metabolic changes associated with the two treatments. The trial has been extended to assess efficacy.

The other important finding in the polyoestradiol study was, as predicted, a protective effect of oestrogen on bone health: 18 serious skeletal events were reported in the group undergoing combined androgen deprivation versus none in the polyoestradiol group (p=0·001).^29^ Osteoporosis and associated events are important and costly side-effects of LHRHa.^7^, ^9^ Strategies to mitigate LHRHa-induced bone loss include use of bisphosphonates, targeting of RANKL (with, for example, denosumab), and use of selective oestrogen-receptor modulators. All these approaches, however, are expensive, but would potentially be unwarranted if an effective method of achieving androgen deprivation without associated bone loss were available. The extended PATCH trial will investigate the effects of LHRHa and oestrogen patches on bone health by assessment of fractures and bone-mineral density.

Our findings that fasting total cholesterol, HDL cholesterol, and fasting glucose concentrations in serum increased in patients receiving LHRHa are in keeping with previous reports.^11^, ^31^ During the first year of treatment with LHRHa, increases of total cholesterol concentrations by 5–10% and of HDL cholesterol by 6–12% have been reported in several small studies.^31^, ^32^, ^33^, ^34^ Observed increases in fasting glucose concentrations by 1–2% have also been seen in short-term (12-week) studies,^35^, ^36^ and larger changes have been seen in one longer-term study.^34^ These results are again consistent with our findings (increases of 2% and 6% in fasting glucose concentrations at 6 and 12 months, respectively). The metabolic abnormalities associated with use of LHRHa have similarities to those in the metabolic syndrome: increases in obesity and concentrations of total and LDL cholesterol and triglycerides, and decreases in lean mass and insulin sensitivity.^11^ The effects with LHRHa, however, also include increases in HDL cholesterol concentrations and subcutaneous fat, but no effect on blood pressure is seen.^37^ The changes we noted in lipid profiles in the oestrogen-patches group are in keeping with the known beneficial arterial effects of oral and parenteral oestrogen therapy,^28^, ^38^ and the decrease in glucose concentrations is consistent with the beneficial effects of oestradiol on pancreatic β-cell function.^39^ A limitation of our analysis, however, is that data on triglycerides and LDL cholesterol were not collected. Changes in concomitant medication that might affect lipid profiles were also not recorded, but, as this was a randomised study, it is likely that these effects would have been balanced across the arms. As the trial was not powered to compare changes in metabolic factors, results should be confirmed in future trials.

Other adverse effects with the two treatments were largely as expected and were generally mild. Notable effects in the oestrogen-patches group were gynaecomastia and minor skin disorders. Fewer episodes of hot flushes were reported than in the LHRHa group. In this population of elderly men, the effects of treatments can be difficult to distinguish from normal signs and symptoms associated with increasing age. Thus, effects on wider features, such as cognition and quality of life in particular, require more detailed investigation.

Oestrogen patches seem to be a potential alternative to LHRHa for men with prostate cancer. Patches offer a low-cost, single therapy that can be self-administered, and which might avoid some of the side-effects associated with LHRHa. Therefore, if shown to be effective in phase 3 trials, oestrogen patches might appeal to patients and clinicians. The observations that oestrogen did not increase either fasting glucose or cholesterol concentrations compared with LHRHa are important and strengthen the rationale for further assessment. For individual patients treatment decisions will need to be balanced and take into account antitumour effects, risks of cardiovascular events, effects on bone health, quality of life, including sexual dysfunction, and possible negative effects on cognition. These latter effects are commonly overlooked when physicians are deciding the best method of achieving androgen deprivation. A large phase 3 study will be required to assess all these features of treatment fully. Overall, though, the need to prioritise the assessment of a potential method of androgen deprivation that could avoid the toxic effects associated with standard therapy, and to avoid the addition of new and expensive agents to counteract them, is clear.
