# Supplementary material for: Cardiovascular outcomes in patients with locally advanced and metastatic prostate cancer treated with luteinising-hormone-releasing-hormone agonists or transdermal oestrogen: the randomised, phase 2 MRC PATCH trial (PR09)
Source: Lancet Oncol. 2013 Apr;14(4):306–16. doi: 10.1016/S1470-2045(13)70025-1 (PMC3620898; doi:10.1016/S1470-2045(13)70025-1)
Supplement: Supplementary appendix [file mmc1.pdf]

THE LANCET Oncology

Supplementary appendix

This appendix formed part of the original submission and has been peer reviewed. We post it as supplied by the authors.

Supplement to: Langley RE, Cafferty FH, Alhasso AA, et al. Cardiovascular outcomes in patients with locally advanced and metastatic prostate cancer treated with luteinising-hormone-releasing-hormone agonists or transdermal oestrogen: the randomised, phase 2 MRC PATCH trial (PR09). *Lancet Oncol* 2013; published online March 4. http://dx.doi.org/10.1016/S1470-2045(13)70025-1.

Appendix Participating Investigators and sites

Investigator	Site	Patients enrolled
Subramanian Kanaga Sundaram	Pinderfields General Hospital	42
Sanjay Dixit	Scunthorpe General Hospital,<br>Diana Princess of Wales Hospital	19
Howard Kynaston	University Hospital of Wales	17
Paul Abel	Imperial College NHS Trust	16
Andrew Robertson	Scarborough General Hospital	16
Abdulla Al-hasso	Beatson West of Scotland Cancer Centre<br>The New Victoria Hospital	15
David Chadwick	James Cook University Hospital	15
Andrew Stockdale	University Hospitals of Coventry and Warwickshire	15
John Hetherington	Castle Hill Hospital	14
Alvan Pope	Hillingdon Hospital	14
Christopher Scrase	Ipswich Hospital	14
Noel Clarke	Hope Hospital	10
Gerald Collins	Stepping Hill Hospital<br>Macclesfield District General	8<br>2
Santhanam Sundar	Nottingham University Hospitals	7
Sharon Beesley	Maidstone Hospital	5
Marc Laniado	Wexham Park Hospital	5
Simon Stewart	St Mary's Hospital, London	4
John Logue<br>James Wylie	Leighton Hospital	3
Simon Williams	London Road Community Hospital<br>Royal Derby Hospital	3
Helen Patterson	Addenbrooke's Hospital	3
Robert Huddart	Croydon University Hospital	3
Roger Kockelbergh	Leicester General Hospital	2
Garret Durkan<br>Edgar Paez	Freeman Hospital	1
Mark Churn	Kidderminster General Hospital	1
